# Oral Findings Linked to Chronic Kidney Disease: A Comprehensive Systematic Review

**DOI:** 10.3390/jcm14124380

**Published:** 2025-06-19

**Authors:** Paula García-Rios, Francisco Javier Rodríguez-Lozano, Nuria Pérez-Guzmán

**Affiliations:** Gerodontologý an Special Care Dentistry Unit, Morales Meseguer Hospital, Faculty of Medicine, IMIB-Arrixaca, University of Murcia, 30008 Murcia, Spain; paula.garciar@um.es (P.G.-R.); nuriap_g@hotmail.com (N.P.-G.)

**Keywords:** chronic kidney disease, oral manifestations, periodontal disease, hyposalivation, saliva, xerostomia

## Abstract

**Background\Objectives:** Chronic kidney disease (CKD) is defined as a clinical syndrome secondary to a permanent change in kidney function or structure, making it irreversible. Most patients at the onset of the disease are asymptomatic or present nonspecific symptoms, including signs and symptoms at the oral level. These manifestations, such as hyposalivation, increased calculus index, enamel defects, or changes in saliva composition, contribute to the diagnosis of this pathology and can also significantly affect the patient’s quality of life. The aim is to systematically assess the presence and relevance of oral manifestations in patients with CKD, and to identify correlations between these symptoms and clinical parameters such as glomerular filtration rate or concomitant conditions of the patient. **Materials and Methods:** A systematic review was conducted following the Preferred Reporting Items for Systematic Reviews and Meta-Analyses (PRISMA) guidelines. A search was carried out in the PubMed, Scopus, Scielo, and The Cochrane Library databases on 7 April 2025, using terms related to “chronic kidney disease” and “oral manifestations”. Inclusion criteria referred to observational studies published in the last ten years that reported oral symptoms in patients with CKD. The quality of cohort and case-control studies was assessed using the Newcastle–Ottawa Scale (NOS), while for cross-sectional studies, the Joanna Briggs Institute (JBI) critical appraisal checklist was used. **Results:** A total of 27 studies met the inclusion criteria, primarily cross-sectional in design. The most frequently reported oral manifestations included hyposalivation, increased calculus and plaque indices, enamel defects, periodontal disease, and oral candidiasis. Significant associations were identified between the duration of dialysis and severity of periodontal disease, as well as between CKD stage and taste dysfunction. Findings varied by age group and CKD stage, with children showing distinct salivary profiles and adults presenting more pronounced periodontal and mucosal conditions. **Conclusions:** This review highlights a clear relationship between CKD and various oral health disturbances, although more studies are needed to better understand oral–systemic interactions in CKD. What is necessary is the establishment of multidisciplinary care approaches.

## 1. Introduction

Chronic kidney disease (CKD) is defined as a clinical syndrome secondary to a definitive change in the function or structure of the kidney and is therefore irreversible. In this systematic review we will differentiate between CKD affecting the adult population and that concerning the pediatric population; in both cases we can state that a patient will be diagnosed with CKD when presenting, in a period equal to or greater than three months, a glomerular filtration rate (GFR) less than 60 mL/min/1.73 m^2^ and/or a GFR greater than 60 mL/min/1.73 m^2^ but with evidence of damage to the renal structure [1]. However, for newborns or infants less than three months old, the requirement that these findings be maintained for a period of three months or more does not apply [2].

Regarding the causes of CKD development in the adult population, it is currently known that diabetes and hypertension are the main causes. These can be accompanied by the presence of chronic glomerulonephritis, the use of anti-inflammatory medications, a high body mass index, or excessive consumption of tobacco and/or alcohol. On the other hand, CKD of genetic origin accounts for a small proportion of total cases; environmental influences appear to play a greater role in increasing susceptibility to this condition [1,3,4].

In contrast, the development of CKD in children is usually due to congenital anomalies of the kidneys and urinary tract, as well as hereditary diseases [5]. There are also associated conditions, such as premature birth with low birth weight or the growing prevalence of childhood obesity.

The incidence and prevalence of CKD is highly variable depending on the country where it is assessed, but several studies state that its prevalence is increasing. If we differentiate the adult population by age and sex, CKD occurs more frequently in woman and in people over 60 years of age [4].

Diagnosing chronic kidney disease based on clinical presentation is difficult, as many individuals are asymptomatic or present with nonspecific symptoms. Therefore, it is commonly detected incidentally through screening tests such as blood tests or urine dipsticks [3]. To diagnose a person with this condition, estimations of glomerular filtration rate (GFR) are used, along with the pathophysiological study obtained through a kidney biopsy.

To identify the types of patients with CKD, the Kidney Disease Improvement Global Outcomes Group (KDIGO) published a classification according to the severity of CKD based on the combination of cause, GFR, and albuminuria. However, the KDIGO classification cannot be applied when discussing CKD in children, as the underlying causes are usually different. In pediatric cases, classification is based on the presence or absence of congenital anomalies of the kidneys and urinary tract. Moreover, the GFR threshold used for adults differs from those used in children, and the evaluation of albuminuria does not provide significant additional benefit in this population [1,2].

The management of patients with CKD is mainly based on three principles: delaying the progression of chronic kidney disease, treating complications related to the condition, and preparing the patient for renal replacement therapy if necessary. The rate of CKD progression depends on both modifiable and non-modifiable factors. Modifiable predictors include hypertension, hyperglycemia, use of nephrotoxic agents, and smoking. Therefore, treatment aimed at slowing disease progression focuses on controlling blood pressure and blood glucose levels, adjusting or avoiding nephrotoxic medications, and smoking cessation. Non-modifiable factors include age, sex, and race.

Another important aspect of this condition is the appearance of complications resulting from it. These include anemia due to decreased erythropoietin production; reduced levels of calcium, phosphorus, and parathyroid hormone, which negatively impact bone health; and metabolic acidosis due to elevated serum bicarbonate levels. In children, complications may also include growth retardation and even neurocognitive development delays [1,4,5].

Most individuals who reach end-stage kidney disease (ESKD) require treatment with hemodialysis or peritoneal dialysis [4].

Lastly, it is worth noting that in recent years, a growing number of cases have been reported of so-called chronic kidney disease of unknown etiology (CKDu)—that is, CKD without diabetes, hypertension, glomerulonephritis, or other apparent causes. It is believed that CKDu may be linked to heat stress, agrochemicals, or contaminated water; however, as its etiology is not yet well defined, specific preventive and therapeutic interventions are lacking [6].

Despite the increasing number of studies exploring the relationship between CKD and oral health, the existing literature remains fragmented, with inconsistent findings regarding the prevalence and severity of specific oral manifestations, such as caries, periodontal disease, and xerostomia. Additionally, previous reviews have often lacked differentiation between pediatric and adult populations or failed to analyze correlations with clinical indicators like glomerular filtration rate or dialysis status. Therefore, a comprehensive and up-to-date systematic review is necessary to synthesize the current evidence, identify gaps, and support the development of multidisciplinary strategies for early diagnosis and holistic care in CKD patients.

This systematic review aimed to present a qualitative synthesis of studies that refer to the oral manifestations of chronic kidney disease (CKD). Specifically, we aimed to establish how CKD affects the oral cavity by determining the oral manifestations most frequently associated with CKD. We also sought to specify the importance of the perception of these oral signs and symptoms as well as their relationships with population groups.

## 2. Materials and Methods

The PRISMA 2020 guide, an acronym for “Preferred Reporting Items for Systematic reviews and Meta-Analyses”, was used to conduct this systematic review. Ref. [7] Additionally, the review was registered in the PROSPERO database (International Prospective Registry of Systematic Reviews) with the registration number CRD420251037809.

For the elaboration of this systematic review, we included articles that responded to our search terms, as well as those published between 2015 and 2025 that provided information on the oral manifestations presented by patients with chronic kidney disease.

On the other hand, we excluded studies that analyzed only the systemic manifestations of this entity and those that did not meet the inclusion criteria. Only studies published in English or Spanish were included due to the language proficiency of the reviewers. Gray literature and unpublished studies were excluded to ensure the inclusion of peer-reviewed research with adequate methodological quality. Studies involving both pediatric and adult populations were included. However, for clarity and relevance, data from pediatric and adult CKD patients were analyzed and presented separately in the results and discussion sections to reflect differences in clinical presentation and oral manifestations.

The inclusion criteria used should follow the PICO model: population/problem (P): patients with chronic kidney disease; intervention (I): not applicable; comparison/control (C): healthy patients; outcome (O): oral manifestations present in CKD patients. Thus, our PICO question is: which oral conditions are most frequently reported in individuals with chronic kidney disease (CKD)?

### 2.1. Search Strategy

The databases used for the exhaustive search of articles were Pubmed, Scopus, Scielo, and The Cochrane Library. These databases were used to identify studies that included important information on oral manifestations of CKD. The search was conducted on 7 April 2025.

The Mesh (Medical Subject Heading) thesaurus was used to obtain search terms. Those referring to the term chronic kidney disease are as follows: “chronic kidney disease”, “chronic renal insufficiency”, “renal insufficiency, chronic”, and “kidney failure, chronic”, while those referring to the term oral manifestations are listed below: “Oral Manifestations”, “Oral Diseases”, “Oral Health”, “Mouth”, “Dental Care”, “Salivation”, “Gingival Diseases”, “Periodontal Diseases”, “Dry Mouth”, and “Candidiasis, Oral”.

The Boolean operators “AND” and “OR” were used to relate the above-mentioned terms to each other. The results obtained from the searches in the databases are reflected in Table 1.

### 2.2. Study Selection

After the search process, the resulting studies were entered into the bibliographic manager Mendeley (Elsevier) to discard duplicates. Subsequently, a first selection process of articles was carried out, considering their title and abstract, as well as compliance with the established inclusion and exclusion criteria. Finally, the selected studies were read and analyzed in full to confirm their eligibility.

### 2.3. Data Extraction

The following distinct categories of each article were considered for data extraction: the author and year of publication, the type of study included, the number and age of participants involved, the manifestations being analyzed, the presence or absence of a comparative analysis between patients with CKD and healthy patients, and the conclusions drawn from the study.

### 2.4. Quality Analysis

This systematic review is composed of 2 case-control studies, 2 cohort studies, and 23 cross-sectional studies. To assess the quality of these articles, two guidelines were used. The first, known as the Newcastle–Ottawa Scale (NOS) [8], was used to evaluate case-control and cohort studies. On the other hand, the critical appraisal tool of the Joanna Briggs Institute (JBI) was used for the assessment of cross-sectional studies. The Newcastle–Ottawa Scale assigns “stars” based on the fulfillment of criteria across three domains, which differ depending on the type of study being evaluated. For case-control studies, the domains are selection, comparability, and exposure, whereas for cohort studies, the domains are selection, comparability, and outcome. Each domain can receive a maximum of one star, except for comparability, which can be awarded up to two stars. Articles that received between 7 and 9 stars were classified as having a low risk of bias; those scoring between 4 and 6 stars were considered to have a moderate risk; and those with scores between 0 and 3 stars were classified as having a high risk of bias. On the other hand, the JBI Critical Appraisal Tool [9] used for evaluating cross-sectional studies defines what observational studies should include, based on the set of recommendations it provides. This tool comprises eight criteria that determine the methodological quality of an article and the extent to which potential biases have been addressed in its design, conduct, and analysis. Each criterion was rated as “yes”, “no”, “unclear”, or “not applicable”. If the studies meet 6 to 8 criteria, they will be evaluated as having a low risk of bias; if they meet 4 or 5 criteria, they will be classified as having a moderate risk; whereas if they meet 0 to 3 criteria, they will be considered as having a high risk of bias. To be included in the review, each study had to meet at least 50% of the established criteria. Two reviewers (PGR and FJRL) independently assessed the studies and established their final scores, which were reviewed for discrepancies. Any disagreements were resolved by consensus. In cases where consensus could not be reached, a third reviewer (NPG) was consulted.

## 3. Results

### 3.1. Study Selection and Flow Diagram

**Figure 1:**
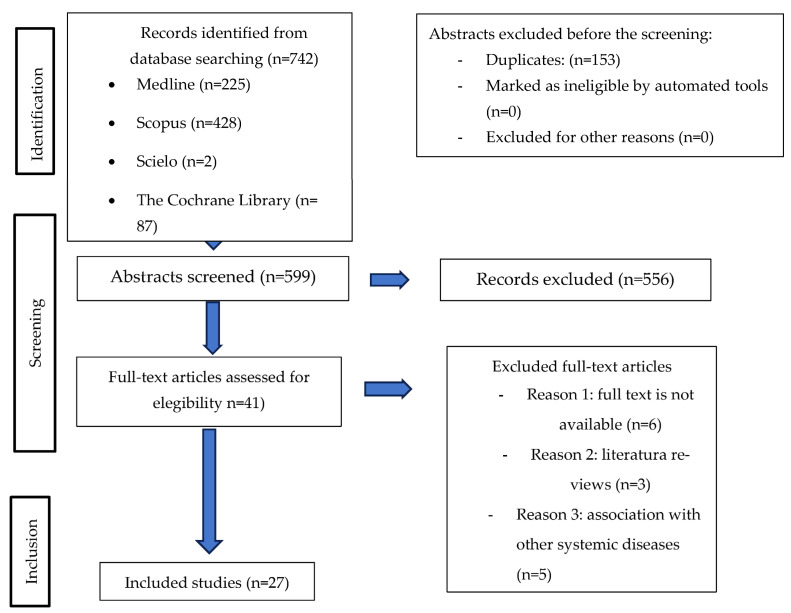
Flow diagram.

The results of the study selection are shown in Figure 1. Through a thorough search in databases, a total of 742 references were found, of which 225 were from Medline PubMed, 428 from SCOPUS, 2 from Scielo, and 87 from The Cochrane Library. Subsequently, 153 duplicate articles were removed using the Mendeley reference manager, leaving 599 articles to be assessed by title and abstract. After this process, 554 references were excluded for not meeting the inclusion criteria, as they focused solely on the systemic symptoms of chronic kidney disease or did not contain data related to oral health. As a result, only 41 articles were read in full, with an additional 14 studies excluded for various reasons: full text not available (*n* = 6), articles classified as reviews (*n* = 3), and associations with other systemic diseases (*n* = 3). A total of 27 studies were selected, as they met all the inclusion criteria and provided comprehensive information on the oral signs and symptoms in patients with chronic kidney disease.

### 3.2. Data Extraction

#### Types of Studies

One cohort study, three case-control studies, and twenty-three cross-sectional studies were included, as shown in Table 2.

### 3.3. Quality Analysis

**Figure 2:**
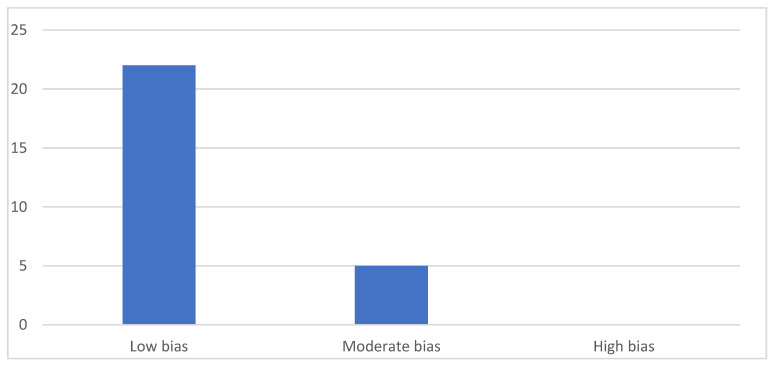
Distribution of studies according to bias.

The results of the quality analysis based on the NOS and JBI guidelines are referenced in Table 3, Table 4 and Table 5, respectively. None of the articles were evaluated as having a high risk of bias; however, five of them were found to present a moderate risk of bias. A summary of the risk of bias distribution is shown in Figure 2.

### 3.4. Bibliometric Analysis

**Figure 3:**
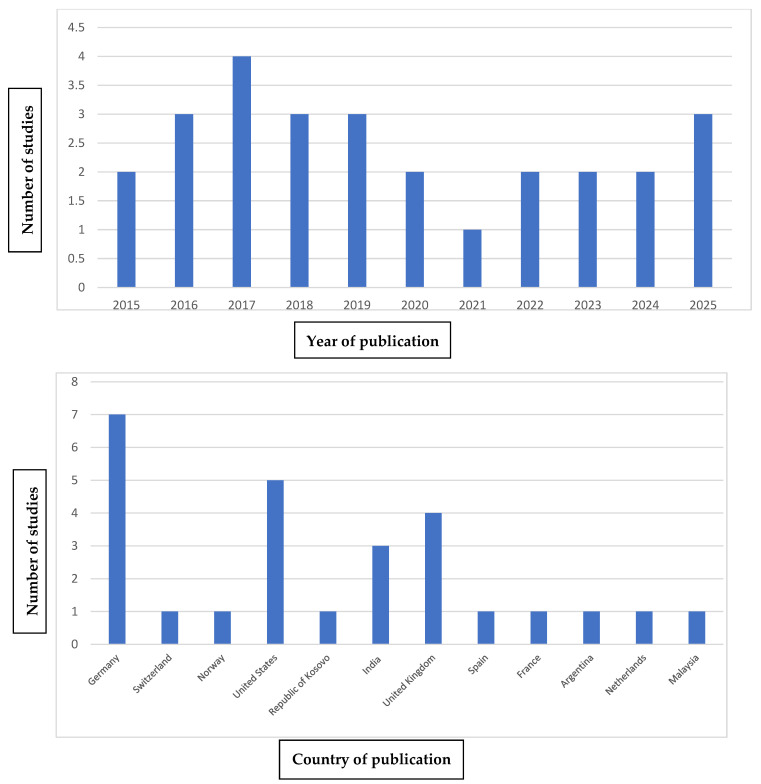

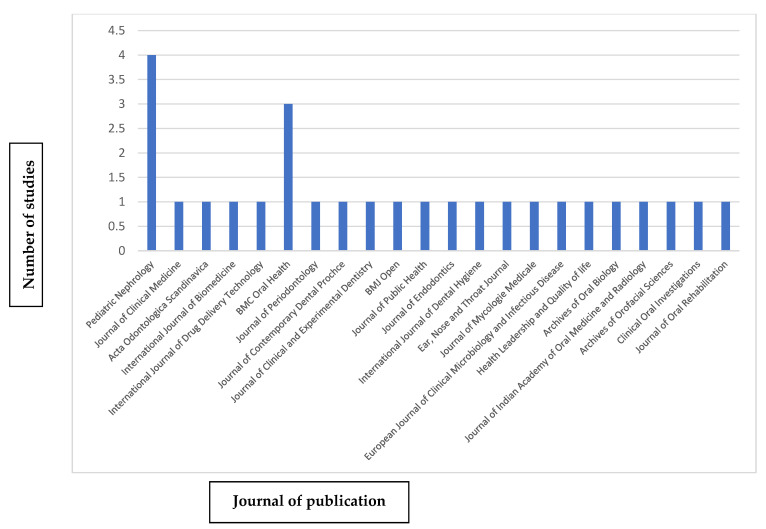
Year, Country and Journal description.

Figure 3 shows the distribution of the articles included in this review based on the year of publication, country, and journal in which they were published.

Regarding the year of publication, it can be observed that articles on this topic have been published every year since 2015. The year with the highest number of publications was 2017, with a total of four studies published.

As for the country of publication, a wide variety of countries where these articles have been published can be noted. In this case, the country with the most publications is Germany.

Like the variety of countries, there is also a wide range of journals in which these articles have been published. Among them, we highlight *Pediatric Nephrology* and *BMC Oral Health*, with four and three publications, respectively.

## 4. Discussion

Chronic kidney disease (CKD) is defined as a clinical syndrome secondary to a permanent change in kidney function or structure; therefore, it is irreversible. In the early stages, patients are usually asymptomatic or present with nonspecific symptoms, which is why diagnosis is often the result of incidental findings during screening tests. This, combined with the increasing prevalence of this condition, makes it difficult to establish an early diagnosis and treatment plan.

This review identified hyposalivation, periodontal disease, increased plaque and calculus indices, and salivary alterations as the most frequently associated oral manifestations in patients with chronic kidney disease (CKD) (Table 6). The analyzed data suggest that factors such as whether the patient is undergoing dialysis, glomerular filtration rate, age, and sex may influence the prevalence of some of the oral manifestations observed in patients with this condition. The hypothesis that CKD is more frequently associated with the appearance of oral signs and symptoms is supported by the findings, although methodological variability in terms of study design or the specific oral manifestation studied highlights the need for further research to confirm these relationships.

This systematic review has been developed with the aim of contributing to quicker diagnosis and treatment plans. It presents and explains the most prevalent oral manifestations in patients with CKD. Oral signs and symptoms are evident and can influence the course of the disease and vice versa. Therefore, it is important to monitor both CKD and its concomitant aspects.

As mentioned at the beginning of this article, to better explain and illustrate the oral manifestation in patients with chronic kidney disease (CKD), we will differentiate the signs and symptoms that appear in the oral cavity based on the age of the patient, that is, distinguishing between children and adults.

We will begin by describing the most common oral manifestations found in children with CKD. For this purpose, we focus on six of the twenty-seven articles included in this review: the studies published by Almeida et al. [10], Dokumacigil et al. [11], Maciejczyk et al. [12], Beyer et al. [13], Caliento et al. [14], and Correa et al. [15] These studies determined that the oral hygiene index (comprising the debris and calculus index) was significantly higher in patients with chronic kidney disease, as well as the presence of salivary metabolites such as creatinine and urea. However, the salivary flow rate was significantly lower in these patients compared to healthy controls. All of this would, theoretically, imply an increase in the presence of caries in children with this condition, but, in contrast, children with CKD showed a significantly lower DMFT index than healthy children. In the study by Almeida et al. [10], which examined a total of 70 children—30 with CKD and 40 healthy children—it was also established that salivary metabolites and their concentrations were clearly different between the two groups. Moreover, the study attributed the lower number of caries found in children with this pathological condition to an increase in salivary pH, a higher buffering capacity of the saliva, and elevated levels of urea and creatinine. This explanation aligns with that provided in the article by Dokumacigil et al. [11], in which a total of 83 children were studied, 40 of whom were healthy. Maciejczyk et al. [12], when examining a group of 60 children—30 of whom had CKD in various stages—added that hyposalivation was more evident in patients with evident stage 4–5 CKD, who were distinguished by their GFR values. A common methodological feature across many studies was the use of the DMFT (Decayed, Missing, and Filled Teeth) index to assess caries experience. While this index offers a general overview, it lacks sensitivity in detecting early or non-cavitated lesions and does not reflect current disease activity. Its widespread use may therefore limit the accuracy of caries prevalence comparisons across studies and populations, particularly in pediatric patients or early-stage CKD cases.

The studies by Beyer et al. [13] and Caliento et al. [14] also noted, in addition to the above, that children with this condition showed a significantly higher prevalence of enamel defects, such as enamel hypoplasia. Furthermore, in Caliento at al.’s article, a higher presence of pale oral mucosa was found in children with this disease. The first study analyzed 167 children, 81 of whom were healthy, while the second had a smaller sample, including only 50 children in the research.

The study by Correa et al. [15] focused on the lingual papillae and the sense of taste. It included a total of 24 children, 12 of whom had CKD, and it was determined that the number and density of fungiform papillae were significantly lower in patients with this disease, with the reduction correlating with decreased GFR. As they have fewer taste papillae, the number of taste receptor cells is also reduced, meaning that taste identification is significantly lower in these patients. All of this was evaluated using a new non-invasive photographic technique.

Now that the most characteristic oral manifestations concerning the pediatric population have been explained, the most prevalent and characteristic oral signs and symptoms in the adult population will be discussed. First, it is important to determine whether there are any differences between men and women regarding the clinical presentation of CKD at the oral level. To this end, the article published by Muhaxheri et al. [16] will be considered. This study involved 90 patients—48 men and 42 women—and established that there are slight differences in the perception of metallic taste and the presence of gingival bleeding, with both being more prevalent in men than in women. Significant differences were found in terms of uremic fetor, which was more frequent in men, while the perception of bad breath and the presence of dental discoloration were significantly more common in women than in men.

On the other hand, unlike the parameters found in the pediatric population, the study by Al-Zaidi et al. [17], which included 60 patients—30 of whom had CKD—found that the prevalence of caries was higher in the group of patients with this condition. Additionally, gingival, plaque, and calculus indices were also higher in CKD patients compared to healthy individuals, although significant differences between the two groups were found in the calculus index.

The studies by Abou-Bakr et al. [18], Han et al. [19], Gupta et al. [20], Ausavarungnirum et al. [21], and Parente et al. [22] determined that one of the most prevalent oral manifestations in patients with CKD was periodontal involvement. In fact, in the study by Abou-Bakr et al. [18], which included 263 CKD patients, the prevalence of periodontitis was found to be 85.6%. A strong positive correlation was observed between patient age and the duration of hemodialysis with increased severity of periodontitis. Moreover, it was established that Stage III periodontitis—characterized by interdental clinical attachment loss greater than 5 mm and the loss of up to four teeth due to the disease—was the most prevalent compared to the other stages (I, II, and IV). This association between periodontal disease and CKD was not only found in cross-sectional studies but was also identified in the cohort study by Han et al. [19], which included a sample of 4,544,610 individuals recruited between 2007 and 2008. The study observed a significant association between age, body mass index, smoking, and end-stage renal disease and the presence of periodontitis. In the study by Gupta et al. [20], the relationship between periodontal conditions in dialysis and pre-dialysis patients was examined. The study included a sample of 90 patients divided into three groups: Group I—30 CKD patients undergoing dialysis; Group II—30 pre-dialysis patients; and Group III—30 healthy individuals. The oral hygiene index, plaque index, probing depth, and clinical attachment level were highest in Group I, followed by Group II. In contrast, the gingival index was highest among healthy individuals. Furthermore, when considering CKD severity, a positive correlation was established with the presence of periodontal disease. In other words, periodontal involvement was more prevalent in patients with more severe CKD compared to those with milder forms of the disease, as concluded in the article published by Ausavarungnirum et al. [21]. It is also important to consider the potential bidirectional relationship between periodontitis and chronic kidney disease. While CKD can lead to immune dysregulation and oral health deterioration, chronic oral infections such as periodontitis may, in turn, contribute to CKD progression through systemic inflammatory pathways. Elevated inflammatory markers associated with periodontitis, such as C-reactive protein and interleukins, have been linked to worsening renal function. This underscores the importance of early detection and management of periodontal disease as part of a multidisciplinary approach to CKD care [21].

Lastly, in the study by Parente et al. [22], it was found that visible plaque and gingival bleeding indices were significantly higher in healthy individuals. However, the presence of gingivitis and periodontitis, tooth loss, probing depth, and clinical attachment loss were greater in patients with end-stage renal disease. These conclusions were drawn from a case-control study involving 45 patients with end-stage renal disease and 26 healthy individuals. End-stage renal disease was not only associated with a higher prevalence of periodontal disease, but also showed a significant relationship with the presence of apical periodontitis, as established in the article published by Khalighinejad et al. [23]

The involvement of periodontal conditions in patients with CKD was not the only oral manifestation observed in these individuals. In the study by Pham et al. [24], which included 220 patients—111 of whom had CKD—it was found that xerostomia, buffering capacity, and salivary pH were significantly higher in CKD patients. Likewise, the DMFT (Decayed, Missing, and Filled Teeth) index was also higher in this group. However, no significant differences were found between CKD and control groups regarding the number of decayed and filled teeth. Additionally, both stimulated and unstimulated salivary flow, as well as creatinine and urea concentrations in saliva, increased proportionally with the severity of CKD. In patients with chronic kidney disease undergoing hemodialysis, in addition to the findings, xerostomia, halitosis, and increased calculus formation were among the most prevalent oral manifestations. Some of these patients also experienced altered taste sensation, pallor of the oral mucosa, and gingival bleeding. All these findings were reported in the study conducted by Honarmand et al. [25], in which 60 patients were examined—30 healthy individuals and 30 with CKD undergoing dialysis. Other relevant oral signs and symptoms associated with chronic kidney disease were evaluated in the studies by Oyetola et al. [26] and Yusuf et al. [27] In the former, abnormal lip pigmentation was identified as the most frequent oral lesion observed in CKD patients, followed by candidiasis, mucosal pallor, and petechial hemorrhages. A correlation was established between glomerular filtration rate (GFR) and the appearance of oral lesions: high GFR was associated with aphthous ulcers, whereas medium-to-low GFR was more commonly associated with oral candidiasis, xerostomia, halitosis, and abnormal lip pigmentation. Yusuf et al. [27], on the other hand, found a significant association between taste dysfunction and chronic kidney disease, which was significantly related to the duration of the disease. However, no significant association was found with age, gender, or the stage of the disease.

Another common oral manifestation in patients with chronic kidney disease (CKD) is the colonization of the oral cavity by *Candida* species. This is discussed in the articles published by Rosa-García et al. [28], Pieralisi et al. [29], and Gonzales et al. [30] In the first of these, which included a sample of 119 CKD patients, it was found that one or more *Candida* species were isolated in 56.3% of the participants, with *C. albicans*, *C. glabrata*, and *C. tropicalis* being the most frequent. Erythematous oral candidiasis was detected on the tongue in 20 of the 119 patients included in the study, all of whom were undergoing hemodialysis. A significant association was found between the presence of oral candidiasis and older age, female gender, smoking, hemodialysis treatment, and the use of removable dental prostheses. Meanwhile, the article by Pieralisi et al. [29] studied 33 CKD patients, 23 of whom presented with oral lesions, with 18 showing coated tongue (*lingua saburral*). Among these 18 patients, 13 had positive cultures for *Candida* on the dorsum of the tongue, with *C. albicans* being the most frequent species. In fact, yeast colonization was significantly more common on the tongue dorsum than in saliva cultures. In addition to the factors mentioned in the study by Rosa-García et al. [28], this study also identified diabetes mellitus and poor oral hygiene as contributing factors. This finding is supported by the article published by Gonzales et al. [30], which studied a sample of 21 patients and found that 81% of those with *Candida* colonization had poor oral hygiene. Beyond the presence of *Candida*, the lingual microbiome of CKD patients differs notably from that of healthy individuals due to colonization by various bacterial species. According to the article by Luo et al. [31], the most abundant bacterial phyla were *Bacillota*, followed by *Pseudomonadota*, with the most prevalent genera being *Streptococcus* and *Neisseria*.

Finally, oral signs and symptoms are also influenced by the stage of CKD in which the patients are found. The oral manifestations in pre-dialysis patients differ from those observed in patients undergoing dialysis or in the post-dialysis follow-up phase. This topic is addressed in the articles published by Marinoski et al. [32], Mahay et al. [33], Kassim et al. [34], and Nylund et al. [35]. In the first of these, after examining a sample of 100 patients—25 of whom were in the pre-dialysis stage—it was found that oral mucosal lesions such as mucosal pallor were more frequently observed in pre-dialysis patients than in those undergoing dialysis. Similarly, xerostomia, dysgeusia, elevated creatinine levels, and uremic odor were also more prevalent in the pre-dialysis group. However, there were no significant differences between the groups regarding the diagnosis of coated tongue. The findings in the article by Mahay et al. [33] align with those reported by Marinoski et al. [32] and further add that the presence of unpleasant taste and burning sensation were more common in pre-dialysis patients than in those receiving dialysis. Moreover, this study did report significant differences in the diagnosis of coated tongue, which was more frequent in dialysis patients. Kassim et al. [34] further contributed to this topic by conducting a study involving 58 patients, 29 of whom were in the pre-dialysis phase. They found that enamel hypoplasia and gingival enlargement were more prevalent during pre-dialysis. Significant differences were also observed in the DMFT index: although the number of carious teeth was higher in healthy individuals, the number of missing teeth was greater in pre-dialysis patients. According to the study published by Nylund et al. [35], after analyzing the findings above, it can be concluded that patients in the dialysis or post-dialysis follow-up phase tend to exhibit better oral health compared to the initial findings observed in the pre-dialysis stage. In the pre-dialysis phase, there was a higher prevalence of calculus, periodontal pockets, missing teeth, and gingival overgrowth. However, the salivary flow rate was higher in pre-dialysis patients compared to those currently receiving or who had previously received dialysis.

While the most prevalent oral manifestations in pre-dialysis patients are already known, it remains to highlight those that are more likely to appear in patients undergoing dialysis. To this end, the article published by Limeres et al. [36] provides valuable insights. In this study, 88 patients were included, 44 healthy individuals and 44 with CKD in the dialysis phase. The study found that the number of missing teeth, creatinine and urea levels, bacterial plaque and calculus accumulation, and periodontal pocket depth were all higher in the dialysis group compared to the healthy controls. On the other hand, salivary volume was significantly lower in CKD patients undergoing dialysis.

This review presents limitations, as do the rest of the published articles. Among them, it is worth noting that not all the selected studies analyze the same oral manifestation, which makes it difficult to compare them. In addition, many studies use the DMFT index (Decayed, Missing, and Filled Teeth) to diagnose caries instead of using the ICDAS (International Caries Detection and Assessment System), which is the recommended method for diagnosing this lesion. Furthermore, only articles published in the last decade were included, with the aim of keeping the review as up-to-date as possible, and only those published in English or Spanish were considered. Additionally, due to the heterogeneity in study design, populations, and outcome measures, a meta-analysis was not feasible, which limits the ability to statistically quantify associations between CKD and specific oral manifestations.

## 5. Conclusions

Based on the findings of this systematic review, it can be concluded that periodontitis, hyposalivation, enamel defects, and candidiasis are among the most frequently observed oral manifestations in patients with chronic kidney disease (CKD). Although these conditions appear more prevalent in CKD patients compared to healthy controls, methodological variability across studies limits the strength of these associations. Future research should focus on well-designed longitudinal studies using standardized diagnostic criteria and consistent oral health assessment tools. Such studies are essential to better understand causal relationships and improve integrated care strategies for individuals with CKD.

## Figures and Tables

**Table 1:** Search strategy.

Databases	Search Field	Results
Medline (Pubmed)	1# “chronic kidney disease”, “chronic renal insufficiency”, “renal insufficiency, chronic”, “kidney failure, chronic”	53,296
	2# “Oral Manifestations”, “Oral Diseases”, “Oral Health”, “Mouth”, “Dental Care”, “Salivation”, “Gingival Diseases”, “Periodontal Diseases”, “Dry Mouth”, “Candidiasis, Oral”	107,559
	**1# AND 2#**	**225**
SCOPUS	1# “chronic kidney disease”, “chronic renal insufficiency”, “renal insufficiency, chronic”, “kidney failure, chronic”	74,795
	2# “Oral Manifestations”, “Oral Diseases”, “Oral Health”, “Mouth”, “Dental Care”, “Salivation”, “Gingival Diseases”, “Periodontal Diseases”, “Dry Mouth”, “Candidiasis, Oral”	159,019
	**1# AND 2#**	**428**
Scielo	1# “chronic kidney disease”, “chronic renal insufficiency”, “renal insufficiency, chronic”, “kidney failure, chronic”	2077
	2# “Oral Manifestations”, “Oral Diseases”, “Oral Health”, “Mouth”, “Dental Care”, “Salivation”, “Gingival Diseases”, “Periodontal Diseases”, “Dry Mouth”, “Candidiasis, Oral”	169
	**1# AND 2#**	**2**
The Cochrane Library	1# “chronic kidney disease”, “chronic renal insufficiency”, “renal insufficiency, chronic”, “kidney failure, chronic”	17,899
	2# “Oral Manifestations”, “Oral Diseases”, “Oral Health”, “Mouth”, “Dental Care”, “Salivation”, “Gingival Diseases”, “Periodontal Diseases”, “Dry Mouth”, “Candidiasis, Oral”	37,524
	**1# AND 2#**	**87**

**Table 2:** Results of manifestations studied.

Author and Year	Type of Study	Number of Participants and Comparison	Age	Manifestations Studied	Conclusions
Almeida et al. (2017) [10]	Transversal	30 patients with CKD (chronic kidney disease) before and after hemodialysis. 40 healthy patients. There was comparison.	10–23	Caries Dental calculus Saliva characteristics	There are significant differences in the salivary composition of patients with CKD treated with dialysis, those without previous dialysis, and healthy patients. Patients with CKD showed a significantly higher amount of dental calculus compared to healthy patients.
Dokumacigil et al. (2025) [11]	Case-control	43 patients with CKD divided into stages (stage 1–3 (*n* = 14) and stage 4–5 (*n* = 29)). 40 healthy patients. There was comparison.	8–17	Caries Dental calculus	The plaque and calculus indices were significantly higher in healthy patients. Salivary flow was lower in patients with CKD. No significant differences were found regarding caries.
Maciejczyk et al. (2020) [12]	Transversal	30 patients with CKD divided into stages (stage 1–3 and stage 4–5). 30 healthy patients. There was comparison.	9–17	Reduction of salivary flow, Xerostomia, Fungal infections, Caries	Salivary flow was significantly lower in patients with CKD, but their salivary pH was significantly higher.
Beyer et al. (2025) [13]	Transversal	86 patients with CKD divided into stage 1–3, stage 4–5, kidney transplant recipient, and nephrotic syndrome. 81 healthy patients. There was comparison.	4–17	Caries, Dental plaque, Enamel development defects	CKD and kidney transplant significantly influence plaque and calculus accumulation, as well as the prevalence of enamel development defects. No significant differences were found regarding caries.
Caliento et al. (2018) [14]	Transversal	25 patients with CKD. 25 kidney transplant recipients, 50 healthy patients. There was comparison.	0–15	Enamel development defects, Xerostomia, Candidiasis, Pale oral mucosa	Transplant recipients showed more oral manifestations than patients with CKD and healthy patients. Patients with CKD had a higher prevalence of pale oral mucosa and enamel hypoplasia than kidney transplant recipients and healthy patients.
Correa et al. (2015) [15]	Transversal	12 patients with CKD. 12 healthy patients. There was comparison.	5–24	Loss of taste	The group with CKD had a significantly lower papillary density and poorer taste sensitivity compared to healthy patients.
Muhaxheri et al. (2023) [16]	Transversal	48 male patients with CKD. 42 female patients with CKD. There was comparison.	>60	Xerostomia, Caries, Halitosis, Gingival bleeding	Men with CKD had a higher prevalence of halitosis, while in women, the presence of stains was more common. There were no significant differences regarding xerostomia or gingival bleeding.
Al-Zaidi et al. (2022) [17]	Transversal	30 patients with CKD. 30 healthy patients. There was comparison.	24–72	Caries, Lost teeth, Filled teeth, Pale oral mucosa, Xerostomia, Candidiasis	The presence of cavities, calculus index, and lost teeth was significantly higher in patients with CKD. No significant differences were observed in the plaque index and gingival index between the CKD group and healthy patients.
Abou-Bakr et al. (2022) [18]	Transversal	263 patients with end-stage renal disease with different stages of periodontitis. There was comparison.	20–70	Periodontitis	There was a significant association between the patient’s age and the duration of dialysis with respect to the stage of periodontitis. No significant association was found between sex and the stage of periodontitis.
Han et al. (2019) [19]	Cohorts	4,544,610 patients with CKD divided according to the number of teeth. There was comparison.	>20	Periodontitis, Lost teeth	The number of lost teeth showed a strong association with end-stage renal disease.
Gupta et al. (2018) [20]	Transversal	30 patients with CKD on dialysis. 30 patients with CKD without prior dialysis 30 healthy patients. There was comparison.	18–70	Periodontitis, Gingivitis, Dental Plaque, Dental Calculus, Xerostomia, Enamel development defects	The periodontal status of patients with CKD was worse compared to healthy patients. For example, significant differences were found in the clinical attachment level parameter, but not in the probing depth.
Ausavarungnirum et al. (2016) [21]	Transversal	46 patients with mild CKD, 48 patients with moderate CKD, 35 patients with severe CKD. There was comparison.	30–86	Caries, Lost teeth, Filled teeth, Periodontitis	Patients with more severe CKD had more severe periodontal disease than those with less severe CKD. The DMFT index (decayed, missing, and filled teeth) and the number of lost teeth were higher in the moderate CKD group than in healthy patients. However, no significant differences were found regarding sex.
Parente et al. (2023) [22]	Case-control	45 patients with end-stage renal disease. 26 healthy patients. There was comparison.	30–50	Gingival bleeding, Dental plaque, Periodontal status	Patients with end-stage renal disease had a significantly higher prevalence of periodontal disease and plaque accumulation.
Khalighinejad et al. (2017) [23]	Transversal	40 patients with end-stage renal disease. 40 healthy patients. There was comparison.	49–64	Apical periodontitis	Apical periodontitis was significantly more prevalent in patients with end-stage renal disease. There were no significant differences regarding age, sex, or smoking habit.
Pham et al. (2018) [24]	Transversal	111 patients with CKD, 109 healthy patients. There was comparison.	30–63	Caries, Xerostomia	Patients with CKD had a significantly reduced salivary flow rate but a higher level of xerostomia. They also had a significantly higher DMFT index.
Honarmand et al. (2017) [25]	Transversal	30 patients with CKD on dialysis. 30 healthy patients. There was comparison.	22–58	Halitosis, Xerostomia, Pale oral mucosa, Gingival bleeding	Halitosis, xerostomia, presence of calculus, and gingival bleeding were significantly more prevalent in patients with CKD than in healthy patients. No significant differences were found regarding the presence of salivary calcium.
Oyetola et al. (2015) [26]	Transversal	90 patients with CKD. 90 healthy patients. There was comparison.	31–65	Candidiasis, Xerostomia, Gingival Bleeding, Periodontitis, Halitosis, Lip pigmentation	The prevalence of oral lesions was significantly higher in subjects with CKD than in healthy patients, including abnormal lip pigmentation, halitosis, periodontitis, and candidiasis. Age and sex were not significantly related to the likelihood of developing oral lesions.
Yusuf et al. (2021) [27]	Case-control	100 patients with CKD. 100 healthy patients. There was comparison.	31–59	Taste dysfunction	The taste dysfunction was significantly correlated with the increased duration of CKD. Neither age nor the stage of CKD were significantly related to the presence of taste dysfunction.
Rosa-García et al. (2020) [28]	Transversal	119 patients with CKD divided into patients with CKD without the presence of candida, with the presence of candida, and with candidiasis. There was comparison.	22–87	Candidiasis, Xerostomia, Pale oral mucosa	The oral cavity of patients with CKD on dialysis is frequently colonized by *Candida* spp. In addition, its presence is more frequent in woman, smokers, and older individuals.
Pieralisi et al. (2016) [29]	Transversal	33 patients with CKD. There was no comparison.	18–75	Candidiasis	Patients with CKD exhibited a high frequency of tongue coating which was frequently colonized by yeasts, especially *C. albicans* and *C. parapsilosis*. There were no significant differences regarding age.
Gonzales et al. (2024) [30]	Transversal	21 patients with CKD. There was no comparison.	29–43	Candidiasis	A significant relationship was found between oral *Candida* and CKD. Its presence is significantly associated with woman, smokers, oral hygiene, and age.
Luo et al. (2025) [31]	Transversal	31 non-diabetic patients on hemodialysis. 29 diabetic patients on hemodialysis. 33 healthy patients. There was comparison.	44–70	Changes in the lingual microbiome	The lingual coating microbiome was different in patients with CKD compared to healthy patients.
Marinoski et al. (2019) [32]	Transversal	50 patients with CKD on dialysis. 25 patients with CKD without prior dialysis. 25 healthy patients. There was comparison.	18–82	Pale oral mucosa, Burning mouth, Xerostomia, Coated tongue, Defects in the oral mucosa	Pre-dialysis patients showed a significantly higher prevalence of oral lesions such as pale mucosa and coated tongue. No significant differences were found related to age or regarding hyposalivation between the pre-dialysis and dialysis groups.
Mahay et al. (2024) [33]	Transversal	150 patients with CKD on dialysis. 150 healthy patients. There was comparison.	55–57	Xerostomia, Halitosis, Pale oral mucosa, Coated tongue, Candidiasis, Defects in the oral mucosa, Gingivitis, Periodontitis	Pre-dialysis patients exhibit a high prevalence of various oral lesions compared to healthy patients. An example would be the presence of coated tongue, candidiasis, or angular cheilitis. There are no significant differences regarding the presence of herpes simplex.
Kassim et al. (2019) [34]	Transversal	29 patients with CKD without prior dialysis. 29 healthy patients. There was comparison.	>18	Candidiasis, Lip pigmentation, Tongue coating, Pale oral mucosa, Xerostomia, Oral ulcers	Pale mucosa is the most common oral manifestation in pre-dialysis patients. Xerostomia, halitosis, and lip pigmentation are also commonly present in these patients.
Nylund et al. (2017) [35]	Transversal	53 patients with CKD. There was no comparison with the other group.	31–86	Dental calculus, Lost teeth, Caries, Filled teeth, Candidiasis, salivary flow	A significant decrease was observed in the total dental index (TDI) and the periodontal inflammatory load index from pre-dialysis to follow-up.
Limeres et al. (2016) [36]	Transversal	44 patients with CKD. 44 healthy patients. There was comparison.	60–70	Lost teeth	The average number of lost teeth was higher in patients with end-stage renal disease (ESRD) on hemodialysis than in the controls.

**Table 3:** Quality assessment of the studies using the adapted version of NOS for cohorts studies.

Cohorts Studies (NOS)	Selection	Comparability	Outcome	Total Score
Han et al. [19]	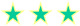	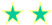	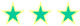	9

**Table 4:** Quality assessment of the studies using the adapted version of NOS for case-control studies.

Case-Control Studies (NOS)	Selection	Comparability	Exposure	Total Score
Dokumacigl et al. [11]	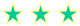	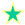	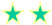	6
Parente et al. [22]	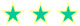	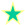	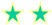	6
Yusuf et al. [27]	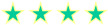	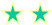	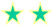	8

**Table 5:** JBI checklist evaluation.

Article Title	Clear Inclusion Criteria	Subjects and Setting Described	Exposure Measured Validly	Standard Criteria for Condition	Confounding Factors Identified	Strategies to Deal with Confounding	Outcomes Measured Validly	Appropriate Statistical Analysis	Overall Appraisal	%
**Almeida et al. (2017)** [10]	Partial	Yes	Yes	Yes	Partial	No	Yes	Yes	Include	**62.5**
**Maciejczyk et al. (2020)** [12]	Yes	Yes	Yes	Yes	Yes	Yes	Yes	Yes	Include	**100**
**Beyer et al. (2025)** [13]	Yes	Yes	Yes	Yes	Yes	No	Partial	Yes	Include	**75**
**Caliento et al. (2018)** [14]	Partial	Yes	Yes	Partial	No	Yes	Yes	Yes	Include	**62.5**
**Correa et al. (2015)** [15]	Yes	Yes	Yes	Yes	Yes	Yes	Yes	Yes	Include	**100**
**Muhaxheri et al. (2023)** [16]	Yes	Yes	Yes	No	Partial	No	Yes	Yes	Include	**62.5**
**Al-Zaidi et al. (2022)** [17]	Yes	Yes	Yes	Yes	Partial	No	Yes	Yes	Include	**75**
**Abou-Bakr et al. (2022)** [18]	Yes	Yes	Yes	Yes	Yes	Yes	Yes	Yes	Include	**100**
**Gupta et al. (2018)** [20]	Yes	Yes	Yes	Yes	Yes	Yes	Yes	Yes	Include	**100**
**Ausavarungnirum et al. (2016)** [21]	Yes	Yes	Yes	Yes	Yes	Yes	Yes	Yes	Include	**100**
**Khalighinejad et al. (2017)** [23]	Yes	Yes	Yes	Yes	Yes	Yes	Yes	Yes	Include	**100**
**Pham et al. (2018)** [24]	Yes	Yes	Yes	Yes	Yes	Yes	Yes	Yes	Include	**100**
**Honarmand et al. (2017)** [25]	Yes	Yes	Yes	Yes	Yes	Yes	Yes	Yes	Include	**100**
**Oyetola et al. (2015)** [26]	Yes	Yes	Yes	Yes	Yes	Yes	Yes	Yes	Include	**100**
**Rosa-García et al. (2020)** [28]	Yes	Yes	Yes	Yes	Yes	Yes	Yes	Yes	Include	**100**
**Pieralisi et al. (2016)** [29]	Yes	Yes	Yes	Yes	Yes	No	Yes	Yes	Include	**87.5**
**Gonzales et al. (2024)** [30]	Yes	Yes	Yes	Yes	Yes	Yes	Yes	Yes	Include	**100**
**Luo et al. (2025)** [31]	Yes	Yes	Yes	Yes	Yes	Yes	Yes	Yes	Include	**100**
**Marinoski et al. (2019)** [32]	Yes	Yes	Yes	Yes	Yes	Yes	Yes	Yes	Include	**100**
**Mahay et al. (2024)** [33]	Yes	Yes	Yes	Yes	Yes	Yes	Yes	Yes	Include	**100**
**Kassim et al. (2019)** [34]	Yes	Yes	Yes	Yes	Yes	Yes	Yes	Yes	Include	**100**
**Nylund et al. (2017)** [35]	Yes	Yes	Yes	Yes	No	Yes	Yes	Yes	Include	**87.5**
**Limeres et al. (2016)** [36]	Yes	Yes	Yes	Yes	Yes	Yes	Yes	Yes	Include	**100**

**Table 6:** Frequency of reported oral manifestations across the 27 included studies. The table summarizes how many studies described each oral condition and the corresponding percentage of the total sample. Some studies reported more than one condition.

Oral Manifestation	Number of Studies Reporting	% of Total (*n* = 27)
Periodontitis	12	44%
Xerostomia/Hyposalivation	11	41%
Candidiasis	9	33%
Enamel defects	5	18%
Halitosis	4	15%
Pale oral mucosa	5	18%
Taste alteration	3	11%
Caries	10	37%
